# Digital epidemiology investments, Saudi Arabia

**DOI:** 10.2471/BLT.25.293876

**Published:** 2026-05-29

**Authors:** Haytham A Sheerah, Ahmed Arafa, Mansour A Alfaya, Ashraf B AlDerbas, Nouf Bin Muammar, Sarah A Alsalhi, Abdulmajeed Y Alahdal, Hessah A Alsalamah, Abdulrahman M Alqahtani, Shada Alsalamah

**Affiliations:** a Population Health Deputyship, Ministry of Health, Riyadh, Saudi Arabia.; b Department of Public Health, Beni-Suef University, Salah Salem Street, 62521 Beni-Suef, Egypt.; c Department of Preventive Medicine, Aseer Health Cluster, Aseer, Saudi Arabia.; d Administration of the Primary Care, Aljouf Health Cluster, Aljouf, Saudi Arabia.; e Department of Family and Community Medicine, King Saud University, Riyadh, Saudi Arabia.; f Preventive Medicine Postgraduate Program, Jeddah First and Second Health Cluster, Jeddah, Saudi Arabia.; g College of Medicine, Almareefa University, Riyadh, Saudi Arabia.; h Computer Engineering Department, Al Yamamah University, Riyadh, Saudi Arabia.; i Information Systems Department, King Saud University, Riyadh, Saudi Arabia.

## Abstract

**Problem:**

Traditional epidemiological surveillance methods are often limited by delays in reporting and fragmented data systems. Saudi Arabia faces additional public health challenges from mass gatherings during Hajj and Umrah, an increasing burden of noncommunicable diseases and rapid urbanization, highlighting the need for investing in digital epidemiology.

**Approach:**

Saudi Arabia has accelerated digital transformation in health care through Vision 2030 initiatives. The strategies include health information exchange platforms, analytics driven by artificial intelligence, telemedicine services and digital monitoring systems used during Hajj. We review current initiatives to invest in digital epidemiology in Saudi Arabia, implementation challenges and policy priorities.

**Local setting:**

Saudi Arabia’s health system operates under a predominantly public model. The health ministry is the main provider, regulator and finance provider of most health-care services. Health-care coverage is nearly universal, with citizens receiving services free of charge through the public system. Ongoing reforms aim to gradually decentralize certain functions.

**Relevant changes:**

The initiatives under Vision 2030 have supported disease surveillance, data integration and public health response capacities. Existing digital health reforms have created a foundation for integrating digital epidemiology into routine public health practice. However, challenges remain, including fragmented interoperability between institutions, workforce shortages, unequal digital access, and concerns about data governance, privacy and algorithmic bias.

**Lessons learnt:**

Saudi Arabia’s experience suggests that digital epidemiology is more effective when integrated within broader digital health reforms. Successful implementation requires not only digital infrastructure, but also workforce development, ethical governance, transparency and mechanisms for integrating digital data into public health decision-making.

## Résumé

**Problème:**

Les méthodes traditionnelles de surveillance épidémiologique sont souvent limitées par des retards dans la transmission des données et par la fragmentation des systèmes de données. L’Arabie saoudite est confrontée à des défis supplémentaires en matière de santé publique liés aux rassemblements de masse lors du Hajj et de la Omra, au fardeau croissant des maladies non transmissibles et à l’urbanisation rapide, ce qui souligne la nécessité d’investir dans l’épidémiologie numérique.

**Approche:**

L’Arabie saoudite a accéléré la transformation numérique dans le domaine des soins de santé grâce aux initiatives de la Vision 2030. Ces stratégies comprennent des plateformes d’échange d’informations de santé, des analyses alimentées par l’intelligence artificielle, des services de télémédecine et des systèmes de surveillance numérique employés pendant le Hajj. La présente étude passe en revue les initiatives actuelles visant à investir dans l’épidémiologie numérique en Arabie saoudite, les défis liés à leur mise en œuvre et les priorités politiques.

**Environnement local:**

Le système de santé saoudien fonctionne selon un modèle majoritairement public. Le ministère de la Santé est le principal prestataire, régulateur et bailleur de fonds de la plupart des services de santé. La couverture des soins de santé est pratiquement universelle, les citoyens bénéficiant de services gratuits au travers du système public. Les réformes en cours visent à décentraliser progressivement certaines fonctions.

**Changements significatifs:**

Les initiatives menées dans le cadre de la Vision 2030 ont favorisé la surveillance des maladies, l’intégration des données et le renforcement des capacités d’intervention de santé publique. Les réformes existantes en matière de santé numérique ont jeté les bases nécessaires à l’intégration de l’épidémiologie numérique dans les pratiques courantes de santé publique. Toutefois, des défis subsistent, notamment le manque d’interopérabilité entre les établissements, la pénurie de main-d’œuvre, les inégalités d’accès au numérique, ainsi que des préoccupations en matière de gouvernance des données, de confidentialité et de biais dû aux algorithmes.

**Leçons tirées:**

L’expérience de l’Arabie saoudite suggère une plus grande efficacité de l’épidémiologie numérique lorsque celle-ci s’inscrit dans le cadre de réformes plus larges en matière de santé numérique. La réussite de la mise en œuvre nécessite non seulement une infrastructure numérique, mais aussi le développement des ressources humaines, une gouvernance éthique, la transparence ainsi que des mécanismes d’intégration des données numériques dans la prise de décision en matière de santé publique.

## Resumen

**Situación:**

Los métodos tradicionales de vigilancia epidemiológica suelen verse limitados por retrasos en la notificación y por sistemas de datos fragmentados. Arabia Saudí se enfrenta además a desafíos de salud pública derivados de las concentraciones masivas de personas durante el Hach y la Umrah, del aumento de la carga de las enfermedades no transmisibles y de la rápida urbanización, lo que destaca la necesidad de invertir en epidemiología digital.

**Enfoque:**

Arabia Saudí ha acelerado la transformación digital de la atención sanitaria mediante las iniciativas de la Visión 2030. Las estrategias incluyen plataformas de intercambio de información sanitaria, análisis basados en inteligencia artificial, servicios de telemedicina y sistemas digitales de vigilancia utilizados durante el Hach. Se examinan las iniciativas actuales de inversión en epidemiología digital en Arabia Saudí, los desafíos para su implementación y las prioridades en materia de políticas.

**Marco regional:**

El sistema sanitario de Arabia Saudí funciona bajo un modelo predominantemente público. El ministerio de salud es el principal proveedor, regulador y financiador de la mayoría de los servicios sanitarios. La cobertura sanitaria es prácticamente universal y la ciudadanía recibe servicios gratuitos a través del sistema público. Las reformas en curso tienen por objeto descentralizar gradualmente determinadas funciones.

**Cambios importantes:**

Las iniciativas de la Visión 2030 han fortalecido la vigilancia de enfermedades, la integración de datos y la capacidad de respuesta en salud pública. Las reformas existentes en materia de salud digital han creado una base para integrar la epidemiología digital en la práctica habitual de la salud pública. No obstante, persisten desafíos, entre ellos la interoperabilidad fragmentada entre instituciones, la escasez de personal, las desigualdades en el acceso digital y las preocupaciones relacionadas con la gobernanza de los datos, la privacidad y los sesgos algorítmicos.

**Lecciones aprendidas:**

La experiencia de Arabia Saudí sugiere que la epidemiología digital es más eficaz cuando se integra en reformas más amplias de salud digital. Su implementación satisfactoria requiere no solo infraestructura digital, sino también desarrollo de capacidades del personal, gobernanza ética, transparencia y mecanismos para incorporar los datos digitales a la toma de decisiones en salud pública.

## ملخص

**المشكلة:**

غالبًا ما تكون طرق الرصد الوبائي التقليدية محدودة بسبب التأخيرات في الإبلاغ وأنظمة البيانات المشتتة. وتواجه المملكة العربية السعودية تحديات إضافية في مجال الصحة العامة نتيجة للتجمعات الكبيرة خلال موسمي الحج والعمرة، وتزايد عبء الأمراض غير المعدية، والتوسع الحضري السريع، مما يُبرز الحاجة إلى الاستثمار في علم الأوبئة الرقمي.

**الأسلوب:**

سارعت المملكة العربية السعودية في التحول الرقمي في مجال الرعاية الصحية من خلال مبادرات رؤية 2030. وتشمل هذه الاستراتيجيات منصات تبادل المعلومات الصحية، والتحليلات المدعومة بالذكاء الاصطناعي، وخدمات العلاج الطبي عن بُعد، وأنظمة المراقبة الرقمية المستخدمة خلال موسم الحج. نستعرض في هذا البحث المبادرات الحالية للاستثمار في علم الأوبئة الرقمي في المملكة العربية السعودية، وتحديات التنفيذ، وأولويات السياسة الحكومية.

**المواقع المحلية:**

يعمل النظام الصحي في المملكة العربية السعودية بموجب نموذج حكومي بشكل رئيسي. وتُعد وزارة الصحة هي المزود الرئيسي، والجهة المنظمة، والممول لمعظم خدمات الرعاية الصحية. وتعد تغطية الرعاية الصحية شاملة تقريبًا، حيث يحصل المواطنون على الخدمات مجانًا من خلال النظام الحكومي. وتهدف الإصلاحات الجارية إلى تطبيق اللامركزية تدريجيًا على وظائف محددة.

**التغيّرات ذات الصلة:**

إن المبادرات بموجب رؤية 2030 تدعم رصد الأمراض، وتكامل البيانات، وقدرات الاستجابة في مجال الصحة العامة. لقد أدت إصلاحات الصحة الرقمية إلى إنشاء أساس لدمج علم الأوبئة الرقمي في ممارسات الصحة العامة الروتينية. ومع ذلك، لا تزال هناك تحديات، تشمل التشغيل المشترك المشتت بين المؤسسات، ونقص الكوادر، والوصول غير العادل إلى البيانات الرقمية، والمخاوف المتعلقة بحوكمة البيانات، والخصوصية، والتحيز الخوارزمي.

**الدروس المستفادة:**

تشير تجربة المملكة العربية السعودية إلى أن علم الأوبئة الرقمي يكون أكثر فعالية عند دمجه ضمن إصلاحات أوسع نطاقًا في مجال الصحة الرقمية. ويتطلب التنفيذ الناجح ليس فقط بنية تحتية رقمية، بل أيضًا تطوير القوى العاملة، والحوكمة الأخلاقية، والشفافية، وآليات دمج البيانات الرقمية في عملية صنع القرار في مجال الصحة العامة.

## 摘要

**问题:**

传统流行病学监测手段常受制于报告滞后与数据系统碎片化的问题。沙特阿拉伯还面临朝觐与副朝期间大型聚众活动、非传染性疾病负担攀升以及快速城市化带来的额外公共卫生难题，凸显出投资发展数字流行病学的必要性。

**方法:**

沙特阿拉伯依托《2030 愿景》(Vision 2030) 相关举措，加快推进医疗健康领域数字化转型。相关举措包括医疗信息交互平台、人工智能驱动的数据分析、远程医疗服务以及朝觐期间启用的数字化监测系统。本文梳理了沙特阿拉伯的当前数字流行病学投资项目、项目实施方面的挑战及政策优先发展方向。

**当地状况:**

沙特阿拉伯的医疗卫生体系以公立运营模式为主。卫生部是大多数医疗护理服务的主要提供方、监管机构和出资主体。医疗卫生保障基本实现全民覆盖，本国公民可通过公立医疗体系免费就医。现行改革旨在逐步下放部分职能。

**相关变化:**

《2030 愿景》框架下的各项举措，助力完善疾病监测、数据整合与公共卫生应急处置能力建设。现有的数字医疗改革为将数字流行病学融入常规公共卫生工作奠定了基础。然而，仍存在一些挑战：医疗机构间数据互通割裂、人才短缺、数字资源获取不均衡，以及数据治理、隐私安全与算法偏见等隐患问题。

**经验教训:**

沙特阿拉伯的实践经验表明，将数字流行病学融入大范围数字医疗改革体系中，其实施成效更佳。实施成功不仅需要数字化基础设施，还需人才队伍建设、合规伦理治理、信息公开制度，以及把数字化数据纳入公共卫生决策的配套机制。

## Резюме

**Проблема:**

Традиционные методы эпидемиологического надзора часто ограничены задержками в предоставлении отчетности и фрагментированностью систем данных. Саудовская Аравия сталкивается с дополнительными проблемами общественного здравоохранения, связанными с массовыми скоплениями людей во время хаджа и умры, растущим бременем неинфекционных заболеваний и стремительной урбанизацией. Все это подчеркивает необходимость инвестиций в цифровую эпидемиологию.

**Подход:**

Саудовская Аравия ускорила цифровую трансформацию здравоохранения в рамках инициатив программы Vision 2030. Среди ключевых направлений – платформы для обмена медицинской информацией, аналитические системы на основе искусственного интеллекта, телемедицинские сервисы и цифровые системы мониторинга, используемые во время хаджа. Авторы рассматривают текущие инвестиционные инициативы в области цифровой эпидемиологии в Саудовской Аравии, проблемы внедрения и приоритетные направления политики.

**Местные условия:**

Система здравоохранения Саудовской Аравии опирается преимущественно на государственную модель. Министерство здравоохранения является основным поставщиком медицинских услуг, регулятором и главным источником финансирования большинства медико-санитарных услуг. Охват медицинской помощью практически всеобщий: граждане получают услуги бесплатно через государственную систему. Текущие реформы направлены на постепенную децентрализацию отдельных функций.

**Осуществленные перемены:**

Инициативы в рамках программы Vision 2030 способствовали развитию эпидемиологического надзора, интеграции данных и укреплению потенциала реагирования системы общественного здравоохранения. Проводимые реформы в области цифрового здравоохранения заложили фундамент для внедрения цифровой эпидемиологии в повседневную практику общественного здравоохранения. Однако сохраняются проблемы, включая недостаточную совместимость информационных систем разных учреждений, нехватку кадров, неравномерный доступ к цифровым технологиям, а также вопросы управления данными, конфиденциальности и алгоритмической предвзятости.

**Выводы:**

Опыт Саудовской Аравии показывает, что цифровая эпидемиология наиболее эффективна, когда интегрирована в более широкие реформы цифрового здравоохранения. Для успешного внедрения необходима не только цифровая инфраструктура, но и развитие кадрового потенциала, этическое регулирование, прозрачность и механизмы интеграции цифровых данных в процессы принятия решений в сфере общественного здравоохранения.

## Introduction

Epidemiology, the core discipline of public health, has traditionally depended on different data collection methods, including population surveys, vital statistics and medical records. These traditional methods have offered important insights into disease trends, risk factors and outcomes. However, these methods are often limited by delays, underreporting and geographic biases, which can slow public health responses, especially during infectious disease outbreaks.^1^

The digital revolution has transformed public health surveillance. The integration of digital technologies such as artificial intelligence (AI), big data analytics (that is, large amounts of highly varied data from many different sources that may be processed rapidly), real-time geospatial mapping, mobile health applications, social media monitoring and wearable biosensors has given rise to the field of digital epidemiology. Unlike traditional methods, digital epidemiology enables continuous, real-time collection and analysis of different data streams, ranging from electronic medical records and pharmacy transactions to social network feeds and smartphone-based symptom checkers. These data sources, when analysed using machine learning algorithms and computational models, facilitate early detection of infectious disease outbreaks, risk stratification and the development of tailored public health interventions.^1^

Saudi Arabia has several distinct public health challenges.^2^^–^^4^ First, the annual Hajj and Umrah pilgrimages bring millions of people from around the world into close contact, heightening the risk of infectious disease outbreaks.^2^ Second, the country faces a growing burden of noncommunicable diseases, including diabetes, obesity, hypertension and cardiovascular disease, which are driven by sedentary lifestyles and dietary transitions.^3^ Third, the rapid urbanization of the Saudi Arabian community has reshaped living conditions, mobility patterns and environmental exposures, creating new public health risks.^4^ These factors not only increase the risk of disease transmission but also demand real-time monitoring, predictive analytics and rapid response capabilities. In this context, this article highlights the rationale for Saudi Arabia’s investment in digital epidemiology and outlines reforms that have created a foundation for integrating digital epidemiology into routine public health practice.

## Local setting

Saudi Arabia’s health system operates under a predominantly public model, with the health ministry serving as the main provider, regulator and financier of most health-care services. The health ministry manages most hospitals and primary health-care centres. Several semi-governmental organizations, such as the National Guard Health Affairs, the Ministry of Defence Health Services and university hospitals, offer services to specific populations. The private sector also plays an increasingly important role, particularly in urban areas, resulting in a mixed health system. The Saudi Arabian government is currently implementing a health-care privatization programme aimed at expanding the private sector’s role in service delivery and reducing the financial burden on the public system. Governance within the Saudi Arabian health system is centralized, with the health ministry and other national bodies, such as the Saudi Health Council and the Public Health Authority, overseeing strategy, regulation and implementation. Ongoing reforms under the Health Sector Transformation Programme aim to gradually decentralize certain functions. Health-care coverage in Saudi Arabia is nearly universal, with citizens receiving services free of charge through the public system, while residents and expatriates are covered through mandatory private health insurance. The system incorporates some co-payment mechanisms in the private sector, mainly for services beyond basic coverage or at premium facilities.^5^^,^^6^

## Approach

Vision 2030, Saudi Arabia’s long-term national strategy, includes the National Digital Transformation Programme, which promotes digital transformation and AI integration across sectors, including health care.^7^ The strategies under Vision 2030 include health information exchange platforms, AI-driven analytics, telemedicine services and digital monitoring systems during Hajj. Saudi Arabia has compelling reasons to invest in digital epidemiology, given its distinct health challenges and evolving population dynamics. With the annual pilgrimages, these digital tools can enable real-time surveillance, rapid contact tracing and crowd health monitoring to prevent and manage such events more effectively. For noncommunicable diseases, digital epidemiology can facilitate continuous monitoring of the risk factors through mobile health applications, wearable sensors and population-level data analytics, which supports early intervention and health promotion. Given the rapid urbanization of the Saudi Arabian population, digital platforms allow integration of environmental, behavioural and clinical data to help policy-makers design healthier and more sustainable urban environments. 

## Relevant changes

In line with Vision 2030, the health ministry established the National Platform for Health Information Exchange Services, which enables seamless data sharing among health-care providers and the National Health Information Center, which oversees the integration of health data and the interoperability of electronic medical records.^8^^,^^9^ Additionally, the Saudi Data and Artificial Intelligence Authority supports big data analytics and AI-driven health-care innovations. The national data bank of this authority consolidates various data sources, including health data, and provides an analytics platform for predictive modelling and disease trend forecasting. *Estishraf*, a predictive analytics platform developed by the Saudi Data and Artificial Intelligence Authority, uses AI to anticipate health-care needs and detect potential outbreaks or health crises, further advancing the capabilities of digital epidemiology.^10^ In addition, the Smart Hajj programme was established to digitize services for pilgrims and has become a model for large-scale public health monitoring. The programme uses digital tools, including mobile applications and connected sensors, to monitor pilgrims’ health during Hajj.^11^ This data-driven approach enables timely identification of health issues, such as respiratory infections or heatstroke, and provides updated information on potential public health risks, which is vital for outbreak prevention.^12^

## Lessons learnt

Existing investments under Vision 2030, including health information exchange systems, AI-driven analytics platforms and digital monitoring tools used during Hajj, have created an important foundation for adopting data-driven public health approaches. However, the implementation of digital epidemiology has faced several challenges. One important barrier is the fragmentation of data systems across different health-care providers and institutions, which limits data integration and interoperability. Another issue is the shortage of specialized professionals, including data scientists, epidemiologists and digital health experts, capable of managing and analysing large, complex data sets. Additionally, data privacy and ethical governance are ongoing concerns. As digital epidemiology relies on personal and location-based data, establishing robust legal frameworks and public trust is essential to prevent misuse and ensure transparency. Other obstacles include limited public awareness of digital epidemiology, uneven digital infrastructure in remote regions and resistance to adopting new technologies. Unequal access to digital technologies may also widen existing health disparities. At the same time, algorithmic bias and overreliance on proprietary or non-health data sources may affect the reliability and fairness of digital epidemiology systems.^13^

To effectively integrate digital epidemiology into Saudi Arabia’s public health system, the development of a comprehensive national digital epidemiology strategy is needed (Box 1). This strategy should prioritize the establishment of a structured framework that guides the adoption of AI, big data analytics and wearable health technologies to track disease trends, predict outbreaks and implement personalized public health interventions. A central aspect of the proposed strategy would be the development of a national repository for epidemiological data that is accessible to researchers and policy-makers across the country.

Box 1 **Pillars of a proposed national digital epidemiology strategy, Saudi Arabia**

**Technology frameworks**

- Establish clear guidelines for adopting AI, big data (large amounts of highly varied data from many different sources that may be processed rapidly) and wearable technology.
- Integrate these tools into existing health systems.
- Standardize protocols for disease tracking and personalized interventions.

**National data repository**

- Create a centralized, accessible epidemiological database.

**AI and data infrastructure**

- Invest in robust infrastructure for data collection, sharing and integration.
- Enable real-time analysis from hospitals, health ministry applications and wearable devices.
- Support predictive modelling for outbreak forecasting.

**Workforce training**

- Train public health professionals on using large language models, machine learning and data analytics.
- Develop specialized programmes in digital epidemiology and health modelling for health professionals.

**International collaboration**

- Partner with global health bodies and experts.
- Align strategies with international standards for global health security.

**Ethical governance and data protection**

- Develop a national framework to ensure ethical data collection, storage and use.
- Enforce privacy, consent and data security regulations.

AI: artificial intelligence.

In parallel, enhancing workforce training is important. Epidemiologists and public health professionals need digital skills to navigate emerging technologies. Specialized training programmes that focus on data analysis, epidemiological modelling and the use of digital health tools will ensure that professionals can effectively use these technologies to monitor health trends, detect outbreaks and implement preventive measures.

Additionally, strengthening international collaborations is an essential step to ensure that Saudi Arabia can learn from best practices and align its strategies with global health standards and thereby remain at the forefront of digital epidemiology.

Integrating ethical and governance considerations is crucial for the responsible implementation of digital epidemiology in Saudi Arabia. Clear policies must safeguard data privacy, security and informed consent, ensuring that health information is used transparently and ethically. Establishing a national ethical governance framework and data protection regulations will help prevent misuse and build public trust. Transparent policies on data collection, sharing and algorithm use, alongside clear accountability and independent oversight mechanisms, are also important to maintain public confidence in large-scale data integration initiatives.

To conclude, an important lesson is that investment in digital infrastructure alone is insufficient (Box 2). Successful implementation also requires workforce training, clear governance frameworks, ethical oversight and transparent mechanisms for integrating digital data into routine public health decision-making. Additionally, digital epidemiology may be particularly valuable in settings with mass gatherings and rapidly changing population dynamics, where timely surveillance and rapid response are essential. Saudi Arabia’s experience may therefore provide useful insights for countries seeking to strengthen digital public health systems or improve preparedness for large-scale public events.

Box 2 **Summary of main lessons learnt**

- Digital epidemiology is most effective when integrated into broader digital health reforms.
- Successful implementation requires infrastructure, workforce training and ethical governance.
- Digital epidemiology is particularly valuable for mass gatherings and rapidly changing population dynamics.
